# Laparoscopy for non-palpable undescended testis: comparing outcomes in syndromic and non-syndromic children

**DOI:** 10.1007/s00383-025-06271-9

**Published:** 2025-12-15

**Authors:** Agnes Raaschou Byström, Nilla Hallabro, Caroline Selin, Magnus Anderberg, Anna Börjesson, Martin Salö

**Affiliations:** 1 Department of Pediatric Surgery, Skåne University Hospital, Lund, Sweden; 2 Department of Clinical Sciences, Pediatrics, Lund University, Lund, Sweden

**Keywords:** Undescended testis, Laparoscopy, Syndrome, Fowler Stephens, Vanishing testis

## Abstract

**Purpose:**

Boys with genetic syndromes have an increased risk of undescended testes (UDT), but laparoscopic findings and outcomes after two-stage Fowler-Stephens orchiopexy (FS2) are rarely reported. This study aims to compare laparoscopic findings and results after FS2 between syndromic and non-syndromic boys.

**Methods:**

A retrospective cohort study on boys with UDT who underwent laparoscopy between 2014 and 2024. Syndromic and non-syndromic patients were compared regarding age at surgery, bilaterality, type of UDT, and atrophy rate after FS2.

**Results:**

In total, 190 boys with 298 testicles underwent laparoscopy. Of these, 42% were found to be intra-abdominal, 23% were vanishing testes and in 22% of cases, a testicular nubbin was found. Bilateral (*p* < 0.001) and intra-abdominal UDT (*p* < 0.01) were significantly more common in children with syndromes, whereas testicular nubbin was less common (*p* < 0.01). The median age at laparoscopy was higher in the group with syndromes. Atrophy occurred in 20% of all testicles undergoing FS2, with no significant difference in outcome between groups (*p* > 0,05).

**Conclusion:**

Syndromic boys present with more severe UDT but achieve comparable outcomes after FS2. Early diagnosis and individualized management are essential to preserve testicular viability in this high-risk group.

## Background

Undescended testis (UDT) affects about 1-4.6% of full-term and up to 30% of preterm boys, with roughly one third of cases being bilateral [1, 2]. After spontaneous descent during the first months of life, the prevalence at one year is around 1-1.5% [1, 3].

The underlying cause of UDT is not fully understood [2]. The condition has a multifactorial origin, involving hormonal, environmental and genetic factors [4, 5]. The main contributors to testicular descent include androgens, insulin-like peptide 3 (INSL3), and the gubernaculum.

Several genetic mutations and syndromes are associated with UDT, such as mutations in INSL3 or the androgen receptor, and disorders of sexual differentiation [6–8]. Other syndromes, including Trisomy 21 and Noonan syndrome, are also linked to an increased risk [7]. Although the prevalence of UDT is higher among children with syndromes, most patients with UDT do not have a genetic disorder [9].

UDT is commonly categorized as palpable or non-palpable [1]. For true non-palpable testes, laparoscopy is recommended to identify intra-abdominal cases, which represent about 50–60% [1]. The reported success rate after FS2 varies between 68% and 93% [10–12], while more recent reviews show more consistent outcomes of 85–89% [13–15].

Data on laparoscopic findings and FS2 specifically in syndromic boys are lacking. These boys frequently present with comorbidities that increase perioperative and anesthetic risks. In many of these syndromes, fertility potential is also reduced due to underlying endocrine or gonadal dysfunction, independent of UDT itself [7, 8].

UDT is a common condition with potential long-term effects on fertility and malignancy risk if left untreated. However, laparoscopic findings and outcomes after FS2 remain poorly defined in syndromic boys. This study aimed to clarify these aspects by comparing characteristics, treatment, and surgical outcomes between syndromic and non-syndromic patients.

## Methods

The study received ethical approval (2010/49 and 2025/00464-01) as well as a local permit from the hospital (KVB 2019/9).

### Study design

The study was a retrospective cohort study including children diagnosed with non-palpable UDT who underwent laparoscopy at a tertiary pediatric surgical center between January 2014 and January 2024. The center provides specialized pediatric urology for an area of around 2 million inhabitants. Patients who underwent the FS2 to correct the UDT were then further analyzed as a subgroup of the larger cohort. All data on the subjects and researched variables were collected from digital patient and surgical journals according to the predefined inclusion and exclusion criteria listed below. In patients with bilateral UDT, data from each testis were treated as a separate case. All children who underwent FS2 had a follow-up 9–12 months after the second operation.

### Inclusion and exclusion criteria

Eligible patients were boys with a preoperative International Statistical Classification of Diseases (ICD-10) codes of Q53.1-2 and documented non-palpable UDT when under general anesthesia, who underwent laparoscopy for non-palpable UDT between January 2014 and January 2024. Children with a confirmed DSD were excluded because their gonadal development and surgical management differ markedly from typical UDT, and inclusion could confound the analysis [2, 7].

### Outcome

The main outcomes studied were (a) type of non-palpable UDT: intra-abdominal, testicular nubbin, vanishing testis, and (b) atrophy rate after FS2. A non-palpable testis was defined as one not found on examination under general anesthesia. Vanishing testis was defined as the finding of blind-ending spermatic vessels at laparoscopy, and a testicular nubbin was defined as a small non-functioning remnant found during surgery. A peeping testis or an inguinal testis (both non-palpable under general anesthesia) was classified together as a fourth category; “other”. All peeping testes were operated on with a one-stage laparoscopy-assisted orchidopexy (without dividing the vessels).

Data on testicular size at surgery and check-up were absent in the vast majority of cases. Hence, the surgeons’ postoperative notes 9–12 months after surgery explicitly stating the presence or absence of atrophy were used to collect this parameter.

### Variables

Independent variables included age at surgery, bilaterality (i.e. two non-palpable testes), and the presence of a genetic syndrome defined as a gene or chromosomal aberration previously known to be pathogenic or associated with comorbidities.

### Statistical analysis

The statistical analyses were performed in IBM SPSS Statistics (Version 29.0.2.0 Armonk, NY: IBM Corp) using non-identifiable patient data. The Chi-squared test was used when comparing two groups with binary variables (side, bilaterality, type of UDT, and atrophy post-surgery) or Fisher’s exact test if *n* < 5 in one group. The distribution of age in all groups was skewed, thus presented as median (min-max) and a non-parametric test (Mann-Whitney U-test) was used when comparing the medians of each group. Although some testes were bilateral, non-paired tests were used. A p-value < 0.05 was considered statistically significant.

## Results

**Fig. 1:**
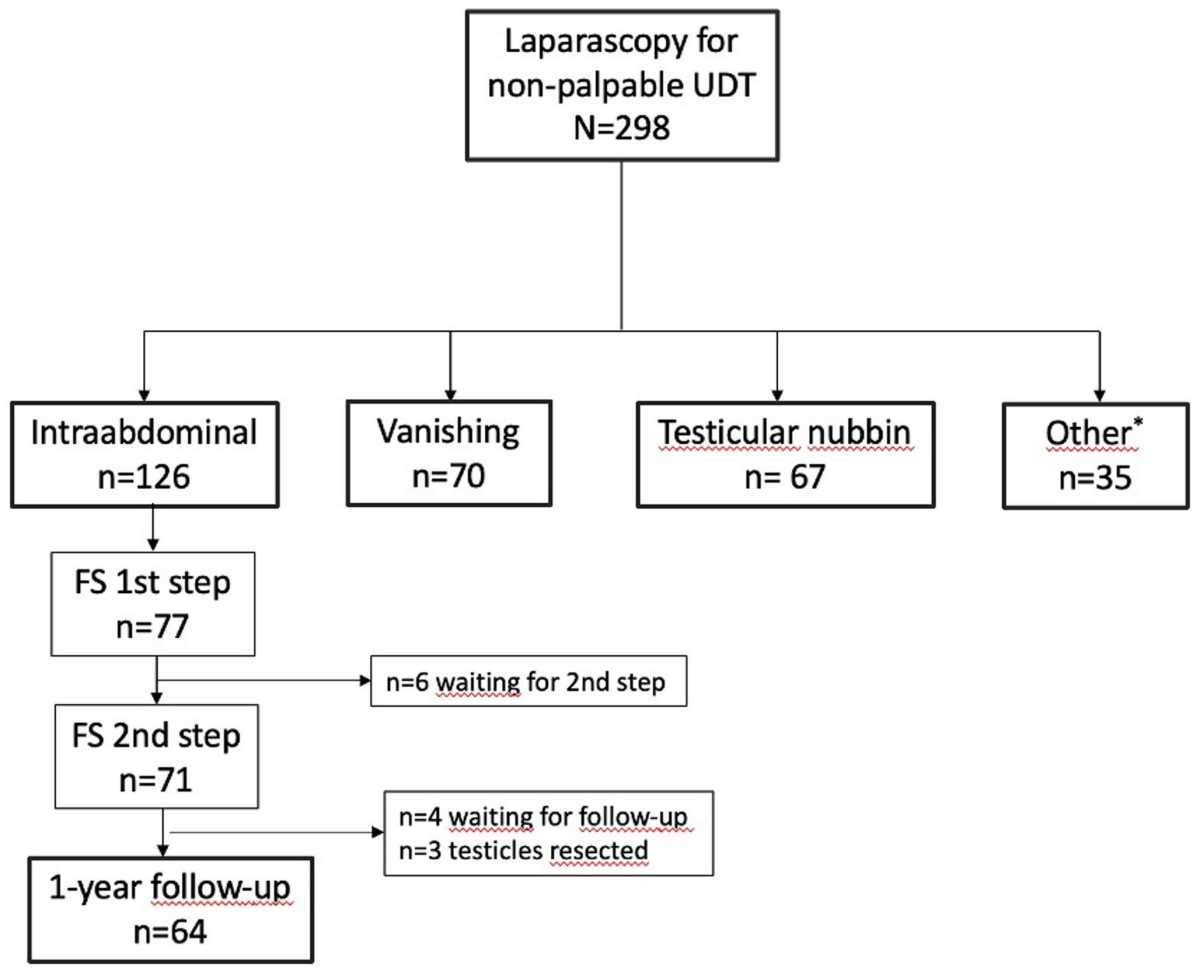
Flowchart showing the inclusion and exclusion of testicles in boys with non-palpable undescended testicles. UDT: undescended testis; FS; Fowler-Stephens; *peeping or inguinal location

During the study period, 190 boys with 298 testes underwent laparoscopy, of which 126 (42%) were found to have a true intra-abdominal UDT at the time of surgery, 70 (23%) were vanishing testes and 67 (22%) were testicular nubbins. There were 35 (12%) testes that were either peeping or eventually identified within the inguinal canal. Of the 126 true intra-abdominal testes, 44 (35%) were operated using laparoscopy-assisted one-stage approach with inguinal dissection, and in five (4%) testes, either no corrective surgery was performed, or details of the procedure were missing from the record. This left 77 (61%) testes that underwent the first step of the FS2 procedure, of which 71 had completed the second surgery. Data from the one-year follow-up was available for 64 testes, constituting the smaller subgroup. Three (4%) testes were removed at the time of the second FS surgery because of atrophy or hypoplastic appearance (Fig. 1).

Among all boys, the median age at laparoscopy was 15 months (0–180 months), 133 (45%) of the testes were on the right side, 54 (18%) were bilateral, and 32 (11%) were found in patients with a known syndrome (Table 1). In total, 21 syndromic patients (32 testes) were included, representing 16 different syndromes (Table 2). Most syndromes were associated with bilateral UDT, whereas eight patients had unilateral involvement.

**Table 1:** Age, laterality, rate of syndromes and intraoperative findings of testicles who underwent laparoscopy for non-palpable undescended testis, and of testicles undergoing two-stage Fowler-Stephens surgery with complete postoperative follow-up

Characteristics	*N* = 298
Age (months)	15 (0–180)
Side (right)	133 (44%)
Bilateral*	54 (18.1%)
Syndrome	32 (10.7%)
*Findings at 1st laparoscopy*	
True intraabdominal testis	126 (42.2%)
Testicular nubbin	67 (22.4%)
Vanishing testis	70 (23.4%)
Other**	35 (11.7%)
*Two-stage Fowler-Stephens*	*N* = 64
Age 1st surgery (months)	17 (9–151)
Age 2nd surgery (months)	25.5 (17–155)
Bilateral***	24 (38%)
Syndrome	16 (25%)

Values presented as median (min-max) and as absolute numbers (percentage), n(%); *only non-palpable bilateral UDT included; **peeping or inguinal location; ***only non-palpable bilateral UDT included

**Table 2:** Syndromic patients with non-palpable UDT who underwent laparoscopy for non-palpable undescended testis

Syndrome	Patients (*N* = 21)	Testes (*N* = 32)
Noonan syndrome	4	7
Denys–Drash syndrome	3	4
Mowat–Wilson syndrome	1	2
4q deletion syndrome	1	2
Nicolaides–Baraitser syndrome	1	2
Osteogenesis imperfecta	1	2
WT1 exon 7 mutation	1	2
TBCD gene mutation	1	2
Trisomy 9 mosaicism with Xq28 duplication	1	2
Prader–Willi syndrome	1	1
Down syndrome	1	1
Cornelia de Lange syndrome	1	1
Ehlers–Danlos syndrome	1	1
Miller–Dieker syndrome	1	1
Limb–Girdle muscular dystrophy	1	1
LH receptor mutation	1	1

Of the 64 testes that underwent FS2 and had follow-up, 24 (38%) belonged to patients with bilateral non-palpable UDT, and 16 (25%) were found in patients with a known syndrome. The median age at the first and second surgeries were 17 (9–151) months and 26 (17–155) months, respectively (Table 1).

At the first laparoscopy, boys with syndromes were older (median 25 months, range 7-173 vs. 15 months, range 0-180; *p* = 0.02), more frequently had bilateral UDT (69% vs. 12%; *p* < 0.001). They also more commonly had true intra-abdominal testes (65% vs. 29%, *p* < 0.01), and less commonly a testicular nubbin (3% vs. 25%, *p* < 0.001), compared to patients without syndromes. No significant differences were found between the groups in side, rate of vanishing testes, or inguinal/peeping testis (Table 3).

**Table 3:** Comparison of age, laterality and findings between testicles in boys with and without syndromes who underwent laparoscopy for non-palpable undescended testis

Characteristics	Syndrome (*N* = 32)	No syndrome (*N* = 266)	*p*-value
Age (months)	25 (7–173)	15 (0–180)	0.02
Side (right)	18 (56%)	115 (43%)	0.16
Bilateral	22 (69%)	32 (12%)	< 0.001
*Findings*			
True intraabdominal testis	21 (65%)	105 (29%)	< 0.01
Testicular nubbin	1 (3%)	66 (25%)	0.12
Vanishing testis	4 (13%)	66 (25%)	< 0.01
Other*	6 (19%)	29 (11%)	0.19

Values presented as absolute numbers (percentage), n(%); and as median (min-max)

*Only non-palpable bilateral UDT included; **peeping or inguinal location

When comparing the 64 cases that underwent FS2 and had complete postoperative follow-up, boys with syndromes were significantly older at the second surgery (median 29 vs. 25 months; *p* = 0.04) and more often had bilateral UDT (69% vs. 24%; *p* < 0.01). No difference was seen in age at the first surgery (17 vs. 16 months; *p* = 0.63). The overall atrophy rate was 20% with no difference between boys with (*n* = 3, 19%) and without (*n* = 10, 21%) a syndrome (*p* = 0.84) (Table 4).

**Table 4:** Comparison of age, laterality, and outcome of testicles in boys with and without syndromes who underwent two stage Fowler-Stephens for non-palpable undescended testis and had complete postoperative follow-up

Two-stage Fowler-Stephens	Syndrome (*N* = 16)	No syndrome (*N* = 48)	*p*-value
Age at 1st surgery (months)	26 (10–151)	15 (9–130)	0.06
Age at 2nd surgery (months)	40 (17–155)	23 (17–136)	0.02
Bilateral*	12 (75%)	12 (23%)	< 0.001
Postoperative atrophy	3 (19%)	10 (21%)	1

Values presented as median (min-max) and as absolute numbers (percentage), n(%);

*Only non-palpable bilateral UDT included

## Discussion

In this retrospective cohort study of the nearly 300 testes operated on laparoscopically, 42% were true intra-abdominal. The rate of true intra-abdominal UDT, bilaterality, and age at surgery were significantly higher, while the rate of residual testicular nubbin was lower among boys with known syndromes. Approximately 80% of the testes had a good outcome after FS2 with no difference between groups.

Of the 298 testicles undergoing laparoscopy 42% were viable and intra-abdominal, a rate within the mid-range of previous reports where the percentage of intra-abdominal UDT varies between 20 and 60% [1, 16–19]. In our study, 35 testicles (12%) were either peeping or actually found viable in the inguinal canal. If these 35 cases were classified as being non-palpable UDTs, the percentage of intra-abdominal UDTs in the present study would be > 50%. Another explanation may be that peeping testis are not classified in a standardized manner, which has also been noted in previous studies [20]. Hence, exact classification of intraabdominal testis varies and therefore the rate. This illustrates that classification differences between studies likely explain much of the variability in reported rates.

When comparing boys with and without known syndromes, the former had a higher rate of true intra-abdominal UDT (65% vs. 29%), a much lower rate of a testicular nubbin (3% vs. 25%), but no significant difference in the rate of vanishing testes (13% vs. 25%). These findings differ notably from studies that did not separate syndromic cases [1, 16–19]. This suggests that the etiology of non-palpable UDT may differ in syndromic children, with a higher prevalence of intra-abdominal location and lower rate of residual testicular tissue. This may reflect a more generalized disturbance of testicular descent, which could also explain the higher rate of bilateral UDT – a notion that has been suggested in previous studies as well [21, 22]. Clinically, this highlights the need for early intervention, since the chance of finding a viable testis appears higher in syndromic children. This is further supported by their comparable FS2 success rates.

Bilateral UDT was significantly more common in syndromic children (69% vs. 12%), and in the FS2 subgroup (75% vs. 23%). This is expected since many syndromes are associated with bilateral UDT, and mutations are more often found in bilateral cases [23] although the exact magnitude of differences has not been well documented. Clinically, this underlines the importance of careful contralateral evaluation, as unilateral UDT or a nubbin is uncommon in this group.

The age at the time of surgery was significantly higher in the laparoscopy-group with known syndromes, 25 vs. 15 months. Similar significant results were found in the groups who underwent FS2, 40 vs. 23 months. The reason for the age discrepancy is unclear, and many possible explanations could be proposed. Comorbidities may increase the frequency of postponed surgeries due to risk of a more severe course of common viral infections. This may postpone the elective UDT surgery several times due to an unacceptable anesthesia risk. Some syndromic children need in-patient care after surgery, which complicate scheduling given limited hospital capacity. We do not believe that the findings reflect lower prioritization of these children for UDT surgery, although this was not specifically studied.

The atrophy rate after FS2 was around 20% in both groups. This is double the previously reported rates of around 8–9% [14, 15]. However, comparisons of atrophy rates between studies are difficult since the definition of post-operative testicular atrophy is not standardized and may vary between studies. Variations may also be seen between different physicians performing the postoperative exam. Another important factor to consider is the rate of testicles being removed up-front and thus never “getting the chance” of an FS2 surgery. In other studies, almost 10% of testicles found in the abdominal cavity are removed [25]. In this study, no testicle was removed at the initial laparoscopy but three were removed during the second stage, possibly already hypoplastic. This may explain the higher atrophy rate. Distinguishing atrophy from, for example, a hypotrophic testicle will be facilitated as time passes and progression or regression in size and viability become more evident [26]. In this study, follow-up was in most cases limited to a single exam around one year, which may under- or overestimate true atrophy rates.

This study adds to the understanding of syndromic non-palpable UDT, describing laparoscopic findings and FS2 outcomes. It also illustrates the distinct characteristics of non-palpable UDT and comparable success rates of FS2 in this group.

## Limitations

Limitations include the retrospective design which allowed no room for more nuanced data to be collected. Testicular size was planned to be assessed but could not be included, as documentation was absent in most cases, despite the fact that documentation of the parameter is recommended. This, together with the lack of consensus on definition of postoperative atrophy, necessitates the use of dichotomous variables [27]. ”It may be considered weak that the defining measure of the primary outcome was the surgeon’s clinical examination at one year follow-up, but this is in accordance with the EAU guidelines (27). All children with suspicion of postoperative atrophy had an inguinal and scrotal ultrasound to confirm the suspicion. Although the overall cohort size was reasonable, the considerably smaller FS2 subgroup - and the even smaller subset of syndromic children - constitutes a substantial limitation. This makes further studies on larger cohorts necessary to draw stronger conclusions. There may also be a risk of ascertainment bias since the frequency of whole genome sequencing probably increased during the decade which this study evaluated. This was partly corrected by checking for syndrome diagnosis after the UDT surgery up until the end of the study period.

With a prospective study design, clear definitions of the included variables could be established beforehand, increasing consistency and reliability. The variables could also be more clearly defined and documented by the physician performing exams or surgery, minimizing subjective interpretation. Since the beginning of this year, a local prospective database of all UDT surgeries including follow-up has been established, with standardized variable definitions.

## Conclusion

This study demonstrates differences in non-palpable UDT characteristics between syndromic and non-syndromic children. Syndromic cases more often presented with true intra-abdominal and bilateral UDTs, yet postoperative outcomes after Fowler-Stephens two-stage orchiopexy were comparable. These findings underscore the importance of early intervention and tailored surgical planning, particularly for syndromic cases, and call for further research to optimize management and long-term outcomes.

## Data Availability

No datasets were generated or analysed during the current study.
