# Correction: Laparoscopy for non-palpable undescended testis: comparing outcomes in syndromic and non-syndromic children

**DOI:** 10.1007/s00383-026-06371-0

**Published:** 2026-02-24

**Authors:** Agnes Raaschou Byström, Nilla Hallabro, Caroline Selin, Magnus Anderberg, Anna Börjesson, Martin Salö

**Affiliations:** 1 Department of Pediatric Surgery, Skåne University Hospital, Lund, Sweden; 2 Department of Clinical Sciences, Pediatrics, Lund University, Lund, Sweden

**Correction: Pediatric Surgery International (2025) 42:41** 10.1007/s00383-025-06271-9

In the original version of the article, the following author name has been incorrectly published.

Incorrect name: Carolin Ericsson Selin.

Correct name: Caroline Selin.

The original article has been updated.

